# A Dosimetric Comparison of the Accumulated Dose in Prostate SBRT for Non-Adaptive and Adaptive External Beam Radiotherapy

**DOI:** 10.3390/cancers18091417

**Published:** 2026-04-29

**Authors:** Richard Lesieur, Sotirios Stathakis, David Solis, Carson Matthews, Krystal Kirby, Christopher William Schneider

**Affiliations:** 1 Department of Physics and Astronomy, Louisiana State University and Agricultural and Mechanical College, 202 Nicholson Hall, Baton Rouge, LA 70802, USA; rlesie1@lsu.edu (R.L.);; 2 Department of Radiation Oncology, Mary Bird Perkin Cancer Center, 4950 Essen Lane, Baton Rouge, LA 70809, USAkkirby@marybird.com (K.K.)

**Keywords:** adaptive radiotherapy, MRI-Guided Adaptive Radiation Therapy (MRgART), prostate SBRT, Image-Guided Radiation Therapy (IGRT), Volumetric Modulated Arc Therapy (VMAT), Intensity-Modulated Radiation Therapy (IMRT)

## Abstract

**Background/Objectives**: Traditional radiotherapy treatments assume that patient anatomy remains unchanged over the course of treatment. Image guidance is used to reproduce the patient setup as closely as possible, and planning margins are used to account for setup errors. With the development of MR-guided Adaptive Radiotherapy (MRgART), daily plan adaptations are now feasible, allowing clinicians to edit the plan according to daily anatomical fluctuations. However, MRgART is currently restricted to step-and-shoot IMRT delivery, which can have reduced dose conformality compared to VMAT. In this study, we compare the accumulated dose over all fractions in prostate SBRT treatments for non-adaptive and adaptive external beam workflows. **Methods**: The simulation and daily images of twenty previously treated MRgART prostate SBRT patients were anonymized. On each simulation image, whole prostate VMAT and MRgART SBRT plans were generated. To simulate non-adaptive treatment dose, the daily images were rigidly registered to the planning images, and the doses were recalculated on the daily images. The MRgART plans were adapted to the daily anatomy and reoptimized. All fractional doses were accumulated, using deformable image registration, and compared to their respective planned doses. **Results**: All MRgART dose accumulations were within clinical tolerance. Four VMAT dose accumulations had a dose constraint that fell outside of clinical tolerance. The VMAT dose accumulations had statistically lower doses to the target compared to their planned doses. **Conclusions**: While high-quality plans can be delivered in a non-adaptive VMAT workflow despite interfractional motion, this study suggests that MRgART produces cumulative dose distributions that more closely resemble the initial treatment plan.

## Simple Summary

Prostate cancer has the highest cancer incidence rate in the United States in males, and approximately 58% of patients receive radiation therapy (RT) sometime during treatment. Conventional RT assumes a relatively static anatomy throughout treatment. However, internal anatomy can move significantly between treatment fractions. With the development of the MRI LINAC and adaptive RT, plan adaptations based upon the patient’s anatomy at the time of treatment are now possible. This retrospective study evaluates how the prostate, bladder, and rectum change in volume and shape between treatment fractions and estimates how the delivered dose deviates from the planned dose for simulated conventional and adaptive RT workflows. We found that the adaptive RT workflow produces more consistent simulated dose accumulations than the conventional RT workflow. This study provides clinicians with a stronger understanding of the dose received by the patient and may lead to adjustments in planning parameters for either workflow.

## 1. Introduction

Prostate cancer has the highest cancer incidence rate in the United States and is the second most frequent cancer in the world among males [1]. Technological developments including advances in imaging and AI-powered techniques have led to improvements in early detection, diagnosis, and treatment [2,3,4]. One of the methods commonly used to treat prostate cancer is external beam radiation therapy (RT), as it is estimated that approximately 58% of prostate cancer patients will undergo radiation therapy at some point in their treatment [5]. In a typical RT workflow, a planning CT of the patient is acquired, followed by treatment planning to deliver a high dose to the target volume while sparing the organs at risk (OARs). Prior to each fraction, image guidance with cone beam CT (CBCT) is used to reproduce the patient setup as closely as possible, and a linear accelerator (LINAC) delivers radiation according to the treatment plan. In this treatment setup, the patient’s anatomy is assumed to be static throughout the treatment process, and errors in the setup are accounted for by adding some margin around the target [6,7,8].

With the recent development of the MRI LINAC and MRI-guided Adaptive Radiation Therapy (MRgART), it is now possible to make plan adjustments based upon the patient’s anatomy immediately prior to the delivery of each fraction [9,10]. The workflow involves taking a planning CT, or in some cases a planning MRI, and optimizing a reference treatment plan to that anatomy. On treatment day, a pretreatment, daily MRI is taken. This provides clinicians with the anatomy on that day. The reference fields are then reoptimized according to changes in the shape and location of the target and OARs before treatment delivery [11]. Plan adaptations are not made when utilizing CBCT for image-guided radiation therapy (IGRT) with a C-Arm LINAC. This may lead to the potential underdosing of the target or overdosing of the OARs due to random or systematic changes in the patient anatomy over the course of treatment [12,13,14].

Although MRgART allows for adjusting the plan according to the patient’s anatomy on the treatment day, there are also limitations associated with this modality when compared with non-adaptive courses of treatment. MRI requires greater imaging time compared to CBCT, and the time spent recontouring and reoptimizing the treatment plan requires the patient to remain in the treatment position significantly longer than for non-adaptive treatments. Furthermore, MRgART is currently limited to gantry static step-and-shoot Intensity-Modulated Radiation Therapy (IMRT) delivery. Volumetric Modulated Arc Therapy (VMAT) has been shown to be capable of delivering superior dose distributions compared to step-and-shoot IMRT [15,16,17]. Since step-and-shoot IMRT requires stopping the beam while MLCs move between control points, the time from the initiation to completion of treatment is longer, increasing the potential for intrafraction motion. Furthermore, the cost of the clinical resources required for implementing and operating an MRgART program remains high.

Due to the increasing use of hypofractionation in the clinic [18], it is critical to have a strong understanding of how target and OAR motion influence the dose delivered to the patient. Several studies have investigated how prostate motion and volume affect the dose during treatment, utilizing various treatment modalities [19,20,21,22,23,24,25]. However, the literature is limited when it comes to comparing MRgART, utilizing step-and-shoot IMRT, and non-adaptive VMAT for the prostate [20,21]. Furthermore, many of the studies have only looked at the per-fraction dosimetric impact, as opposed to the accumulated dose over all fractions. A better understanding of the total accumulated dose a patient may receive from both modalities is important, as any dosimetric differences caused by target or OAR interfractional motion could potentially offset each other over the course of a fractionated treatment regime [26]. It is important for clinicians to understand how the dose may vary from that planned at both over a single fraction and over the accumulated dose from all fractions for both modalities, enabling more informed decisions for each patient and their clinic. To our knowledge, there has been no study that compares the total accumulated dose over all fractions in prostate SBRT between MRgART and non-adaptive VMAT.

This work evaluates how the interfractional volume and shape of the prostate, bladder, and rectum change over the course of five treatment fractions and how the simulated accumulated dose to the patient differs from the originally planned dose for simulated MRgART and non-adaptive prostate SBRT treatments. In addition, the accumulated doses over all fractions are compared between MRgART and non-adaptive VMAT to determine the dosimetric differences between the two modalities.

## 2. Materials and Methods

### 2.1. Patient Cohort and Plan Processing

Twenty patients who had previously undergone five fractions of prostate SBRT on the Elekta Unity MRI LINAC (Elekta AB, Stockholm, Sweden) at Mary Bird Perkins Cancer Center were selected for this study. Patients with medical implants in the pelvic region were not included in this study due to the susceptibility artifacts produced in the daily MRI. The simulation MRI (MAGNETOM Sola, Siemens Healthineers, Erlangen, Germany) and daily pretreatment MRIs were gathered from each patient and anonymized in MIM v7.4.5 (MIM Software Inc., Beachwood, OH, USA). The use of this data and its analysis were approved by the institutional review board IRBAM-25-0232.

All anonymized MRIs were contoured using TheraPanacea’s ART-Plan software package v2.3.2 (TheraPanacea, Paris, France) [27,28]. Pseudo-CTs were generated from all simulation and daily MRIs, using a commercially available MRI-to-pseudo-CT software, TheraPanacea ART-Plan v2.3.2 [29]. The AI-generated contours and pseudo-CTs were visually inspected and verified before continuing. The autogenerated contours were manually edited in cases where there appeared to be discrepancies between the autogenerated contour and the apparent organ boundary. The structures on the MRIs were transferred to their respective pseudo-CTs in MIM v7.4.5, and all contoured pseudo-CTs were imported to Monaco TPS v6.2 (Elekta AB, Stockholm, Sweden) for plan optimization and dose calculation.

### 2.2. Volume and Shape Measurements

To estimate interfractional organ motion, organ-specific volumes and DICE scores were recorded and calculated for the prostate, bladder, and rectum. To accomplish this, rigid registrations of each daily MRI to the corresponding simulation MRI were created using MIM v7.4.5’s point-based image registration tool to align the volumetric center of the prostate on both images. No rotations were included in these registrations. This rigid, point-based registration method simulates the best achievable alignment for a CBCT-guided non-adaptive VMAT workflow utilizing a standard 3-degree-of-freedom (DOF) couch. Fiducial markers are often used in practice to assist with alignment in CBCT-guided prostate SBRT [30,31]. Such fiducial markers were not present in this cohort of patients, but the superior soft tissue contrast provided by MR guidance allowed for accurate localization without the need for fiducials [32].

After registration, the contours from the daily MRIs were transferred to the simulation MRI, and DICE scores were calculated in MIM v7.4.5 for the prostate, bladder, and rectum over each fraction. The mean DICE score and volumes were calculated for each of the five fraction treatment courses. The mean volumes over the five treatment days were compared to the reference volumes on the simulation image, and the percent change from the reference of each structure’s volume over the course of the five fractions was recorded. Tukey’s IQR method was used to identify any outliers in the mean DICE scores or volume differences, indicating larger than normal structure variation.

### 2.3. MRgART Plan Simulation

The MRgART plans were generated in Monaco TPS v6.2 for the Elekta Unity (Elekta AB, Stockholm, Sweden), which uses a 7 MV photon beam. All 20 simulated plans consisted of a five-fraction SBRT treatment delivering 40 Gy to the clinical target volume (CTV), defined as the whole prostate without seminal vesical or lymph node involvement. Each plan consisted of 19 step-and-shoot IMRT fields. The planning target volume (PTV) margins were a uniform expansion of 4 mm around the prostate, except in the posterior direction along the rectum, where margins were reduced to 3 mm. All plans were optimized using the same scorecard dose volume histogram (DVH) goals, and if the primary dose objective could not be achieved, secondary goals were used to maintain clinical acceptability. The scorecard goals are presented in Table 1.

After the optimization of the reference plans, the daily adapted plans were generated using the Adapt to Shape (ATS) workflow. This workflow reoptimizes the beam’s MLC positions and monitor units according to the patient’s recontoured anatomy for that day using the reference plan’s original objectives and constraints. To account for the limited time to optimize the plan due to the patient being on the table, the optimizer was only run once for each daily image.

### 2.4. Non-Adaptive VMAT Plan Simulation

The non-adaptive VMAT plans were generated in Monaco TPS v6.2 for the Elekta VersaHD LINAC (Elekta AB, Stockholm, Sweden). All 20 simulated plans consisted of a five-fraction SBRT treatment delivering 40 Gy to the CTV, defined as the whole prostate without seminal vesical or lymph node involvement. Each plan used two 6 MV flattening filter-free (FFF) VMAT arcs. The PTV margins and scorecard goals were kept the same as those used in the MRgART plans found in Table 1.

After each VMAT plan was optimized on the simulation MRI’s pseudo-CT, the plan’s beams were registered to the center of the prostate on each daily MRI’s pseudo-CT. This alignment simulates the best achievable treatment beam alignment to the target for traditional CBCT with a standard 3 DOF couch. Once registered, the beam was subsequently recalculated on the daily MRI’s pseudo-CT, preserving the original control points and arc weights contained within the planned beam. Because the beams were aligned to the center of the prostate in each fraction and no beam optimization was performed between fractions, the non-adaptive CBCT-guided VMAT SBRT dose was simulated.

### 2.5. Dose Accumulation and Analysis

To estimate the total dose delivered to the anatomy over all five fractions, the simulated doses for both the VMAT and MRgART workflows were imported to MIM v7.4.5 for deformable image registration (DIR) and dose accumulation. A backward dose accumulation strategy was used, which maps fractions two through five back to fraction one using DIR [25,33]. The registration process starts with a rigid registration for initial alignment followed by a hybrid DIR, which uses both contour information and voxel intensity to guide the deformation field [34]. To limit dosimetric errors related to image registration, the DIRs were verified both visually and quantitively, including geometric and dosimetric metrics, before proceeding with the dose accumulation. Visual verification was done by reviewing the anatomical alignment between the original and registered images. Following visual verification, quantitative verification was done by deforming that fraction’s dose and contours to the first fraction’s image and comparing the resulting dose and contours to the native dose and contours found on the fraction’s original image. If the contours were within AAPM’s TG-132 metrics for DICE and MDA [35] and if the dose did not deviate by >1% in PTV_V36.25 Gy_, PTV_D0.03 mL_, and rectum_V36 Gy_ for both workflows, the DIR was accepted. The per-contour DIR QA data is shown in Table 2, and the per-patient per-contour DIR QA data is shown in Table S1. Table 3 shows the dosimetric DIR QA data for each analyzed DVH point for both workflows, and although not used for the QA of the DIRs, Table S2 shows the dosimetric difference data from DIR for all scorecard DVH points. Once all the deformation vector fields were established, the dose distributions from each of the five fractions were transferred to fraction one, scaled to one-fifth of their original dose, and summed. This process resulted in one MRgART and one non-adaptive VMAT dose accumulation for each initial plan.

**Figure 1:**
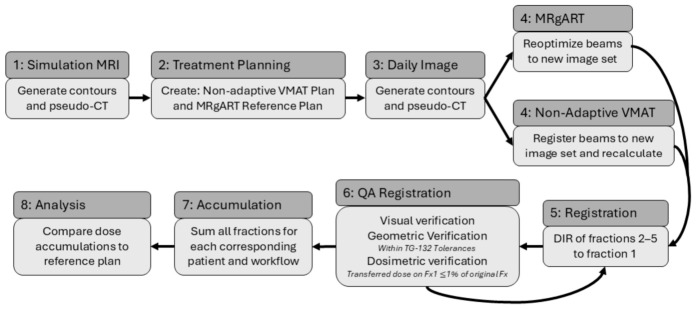
This workflow diagram illustrates the procedure used to generate, accumulate, and analyze dose distributions for adaptive step-and-shoot IMRT and non-adaptive VMAT treatments.

The dose accumulations were analyzed using the scorecard criteria shown in Table 1. Paired Wilcoxon Signed Rank tests were used to compare the median scorecard values for each DVH criterion on the accumulated and planned doses. Specifically, statistical comparisons were made between the planned MRgART and planned VMAT doses; accumulated MRgART and accumulated VMAT doses; the reference MRgART and accumulated MRgART doses; and the reference VMAT and accumulated VMAT doses. To account for multiple testing, a Bonferroni correction was applied, so a *p*-value < 0.0125 was considered statistically significant. A general workflow is shown in Figure 1.

## 3. Results

### 3.1. Prostate, Bladder, and Rectum Volumes and DICE Scores

**Figure 2:**
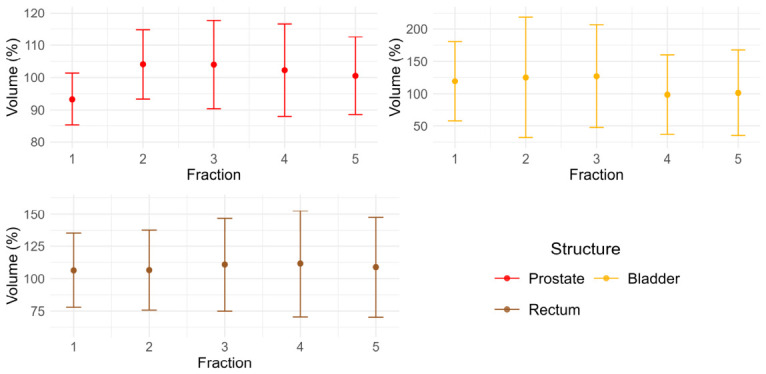
The volumes of the prostate (red), bladder (yellow), and rectum (brown) normalized to the volume at reference. The error bars are 1σ from the mean.

The difference between the prostate volume in the reference image and the mean prostate volume over the treatment days ranged from 0.3 mL to 11.6 mL. The mean (σ) normalized prostate volume over fractionation was 100.9% (10.6) relative to the reference volume, reaching a maximum on the second fraction of 104.1% (10.7) and a minimum on the first fraction of 93.3% (8.1). The difference between the bladder volume in the reference image and the mean bladder volume over the treatment days ranged from 8.6 mL to 204.6 mL. The mean (σ) normalized bladder volume over fractionation was 114.3% (53.3) relative to the reference, reaching a maximum on the third fraction of 127.1% (79.5) and a minimum on the fourth fraction of 98.7% (61.6). The difference between the rectum volume in the reference image and the rectum volume over the treatment days ranged from 0.5 mL to 52.8. The mean (σ) normalized rectum volume over fractionation was 108.9% (28.9) relative to the reference, reaching a maximum on the fourth fraction of 111.6% (40.1) and a minimum on the first fraction of 106.4% (28.6). There were two outliers in the prostate mean volume differences from the reference, no outliers in the bladder mean volume differences, and six outliers in the rectum mean volume differences, according to Tukey’s outlier test. Figure 2 depicts the percent change in volume from the reference volume for the prostate, bladder, and rectum over each fraction. The error bars are 1σ from the mean volume at that fraction and represent the variation in how the prostate changes in volume from the reference volume between patients.

**Figure 3:**
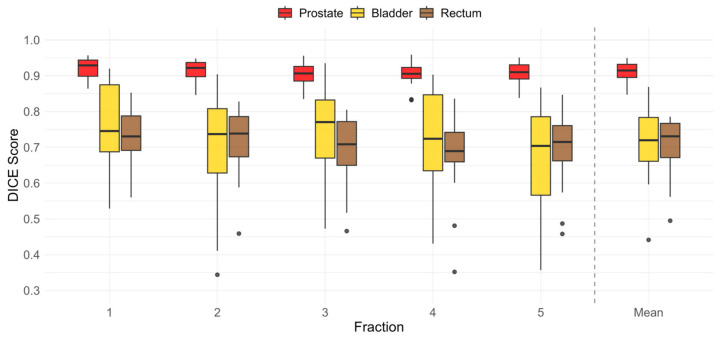
Box and whisker plots of the prostate (red), bladder (yellow), and rectum (brown) DICE scores for each fraction and the mean DICE scores for all fractions.

The largest mean (σ) DICE score for the prostate was 0.9498 (0.0055), and the smallest was 0.8472 (0.0142). The largest mean (σ) DICE score for the bladder was 0.8690 (0.0467), and the smallest was 0.4414 (0.0940). The largest mean (σ) DICE score for the rectum was 0.7778 (0.0494), and the smallest was 0.4950 (0.0435). There were no outliers in the prostate’s mean DICE scores, one outlier in the bladder’s mean DICE scores, and one outlier in the rectum’s mean DICE scores, according to Tukey’s outlier test. Figure 3 depicts the structure DICE scores for each fraction relative to its corresponding structure on the reference image and shows the mean DICE score over all five fractions.

### 3.2. Reference Planning for MRgART and Non-Adaptive VMAT

All MRgART reference plans were deemed clinically acceptable. The rectum D_0.5 mL_ achieved primary goals in 17 plans and secondary goals in three plans. The rectum V_36 Gy_ achieved primary goals in five plans and secondary goals in 15 plans. The rectum V_32.6 Gy_ achieved primary goals in 18 plans and secondary goals in two plans. All other constraints achieved primary goals in all plans.

**Figure 4:**
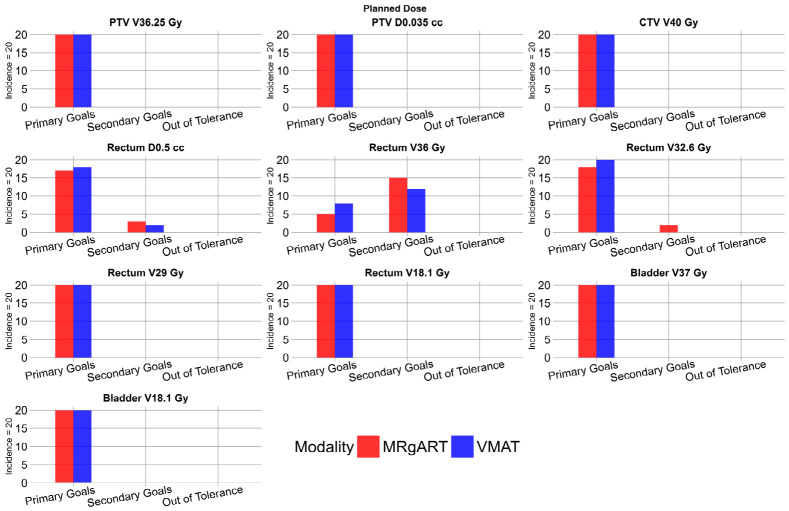
Histograms depicting the incidence of planned doses that met primary goals, met secondary goals, or were out of tolerance, according to scorecard DVH criteria. MRgART plans are shown in red, and VMAT plans are shown in blue.

All non-adaptive VMAT planned doses were deemed clinically acceptable. The rectum D_0.5 mL_ achieved primary goals in 18 plans and secondary goals in two plans. The rectum V_36 Gy_ achieved primary goals in eight plans and secondary goals in 12 plans. All other constraints achieved primary goals in all plans. Figure 4 shows the number of plans achieving primary and secondary goals for each scorecard criterion defined in Table 1, excluding femur heads, in the planned dose for the 20 MRgART and non-adaptive VMAT plans.

### 3.3. Fractional Doses for MRgART and Non-Adaptive VMAT

**Figure 5:**
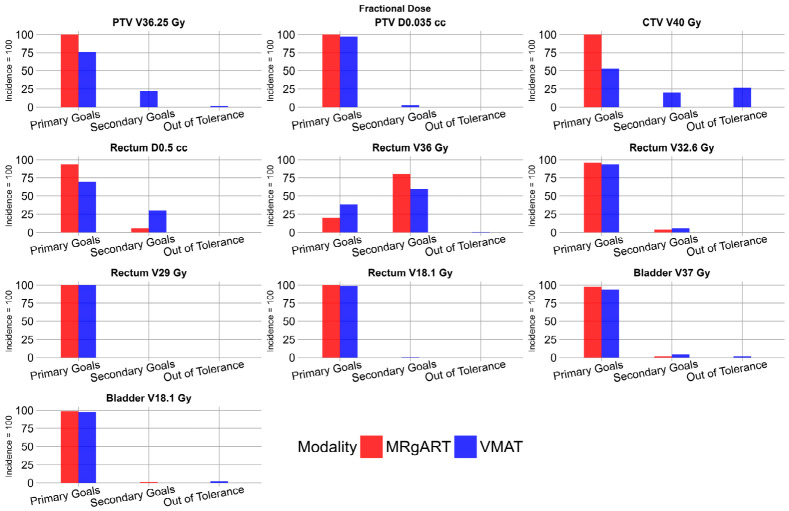
Histograms depicting the incidence of fractional doses that met primary goals, met secondary goals, or were out of tolerance, according to scorecard DVH criteria. MRgART plans are shown in red, and VMAT plans are shown in blue.

In the 100 fractional doses across all 20 patients, all MRgART plans were deemed clinically acceptable, and all fractions achieved primary goals in the PTV and CTV structures. The VMAT fractional doses had 29 cases where at least one DVH constraint was out of tolerance. Figure 5 shows the number of plans achieving primary and secondary goals for each scorecard criterion defined in Table 1, excluding femur heads, in the fractional dose for the 100 MRgART and non-adaptive VMAT plans.

### 3.4. Accumulated Doses for MRgART and Non-Adaptive VMAT

All MRgART dose accumulations were deemed clinically acceptable. The rectum V_36 Gy_ achieved primary goals in six plans and secondary goals in 14 plans. The rectum V_32.6 Gy_ achieved primary goals in 18 plans and secondary goals in two plans. The rectum V_18.1 Gy_ achieved primary goals in 19 plans and secondary goals in one plan. All other constraints achieved primary goals in all plans.

**Figure 6:**
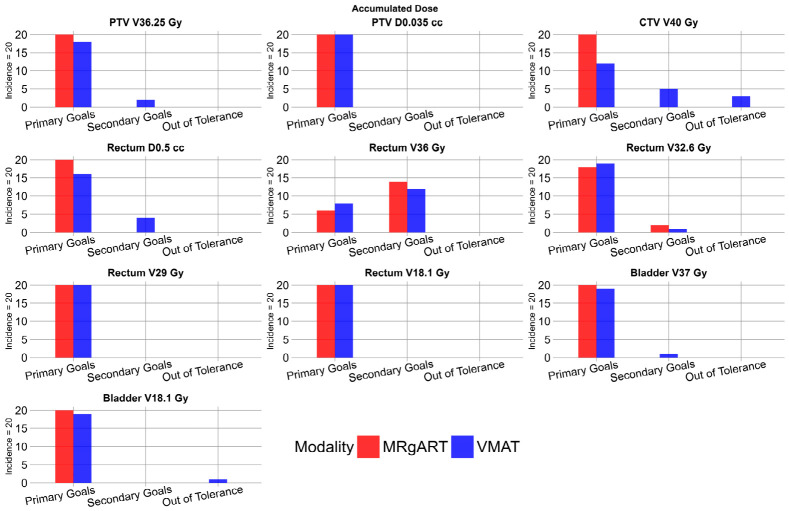
Histograms depicting the incidence of accumulated doses that met primary goals, met secondary goals, or were out of tolerance, according to scorecard DVH criteria. MRgART plans are shown in red, and VMAT plans are shown in blue.

Sixteen non-adaptive VMAT accumulated doses were deemed clinically acceptable, and four non-adaptive VMAT accumulated doses had one DVH constraint outside of clinical tolerance. The PTV V_36.25 Gy_ achieved primary goals in 18 plans and achieved secondary goals in two plans. The CTV V_40 Gy_ achieved primary goals in 12 plans, achieved secondary goals in 5 plans, and was outside of clinical tolerance in 3 plans. The rectum D_0.5 mL_ achieved primary goals in 16 plans and secondary goals in four plans. The rectum V_36 Gy_ achieved primary goals in eight plans and secondary goals in 12 plans. The rectum V_32.6_, rectum V_18.1_, and bladder V_37_ achieved primary goals in 19 plans and secondary goals in one plan. The bladder V_18.1_ achieved primary goals in 19 plans and was out of tolerance in one plan. All other constraints achieved primary goals in all plans. Figure 6 shows the number of plans that achieved primary goals and secondary goals and were out of tolerance for each scorecard criterion defined in Table 1, excluding femur heads, in the accumulated doses for the 20 MRgART and non-adaptive VMAT plans.

### 3.5. Statistical Analysis

The VMAT planned doses had statistically lower OAR doses than the MRgART planned doses for the rectum V_36 Gy_, V_32.6 Gy_, and V_29 Gy_; bladder V_37 Gy_ and V_18.1 Gy_; and the femur head left and right D_1 mL_. The PTV and CTV doses, as well as all other OAR dose constraints, were not statistically different between the two modalities’ planned doses. The MRgART dose accumulations had statistically higher PTV V_36.25_ and prostate V_40 Gy_, as well as statistically lower PTV D_0.035 mL_, than the non-adaptive VMAT dose accumulations. For the OARs, the MRgART dose accumulations had statistically higher rectum V_32.6 Gy_, V_29 Gy_, and V_18.1 Gy_; bladder V_18.1 Gy_; and femur head left D_1 mL_ than the VMAT dose accumulations. Table 4 shows the results of the Wilcoxon Signed Rank tests comparing the MRgART planned and accumulated doses to the VMAT planned and accumulated doses as well as the median DVH values across the 20 simulated treatments. Statistical significance is indicated by bold *p*-values.

**Figure 7:**
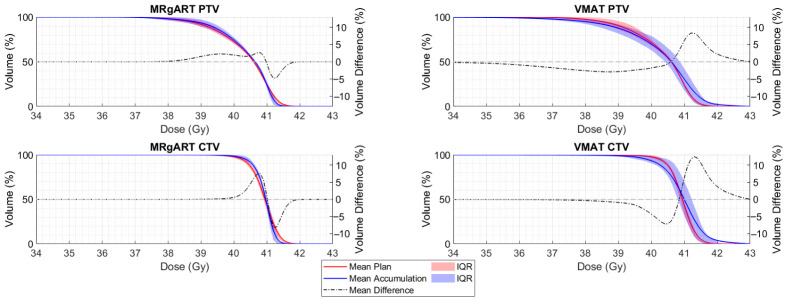
Statistical DVH curves, showing the mean plan and accumulation and their IQR for the PTV and CTV structures in the MRgART and VMAT workflows. The mean difference between the accumulated and planned doses is plotted against the right y-axis.

**Figure 8:**
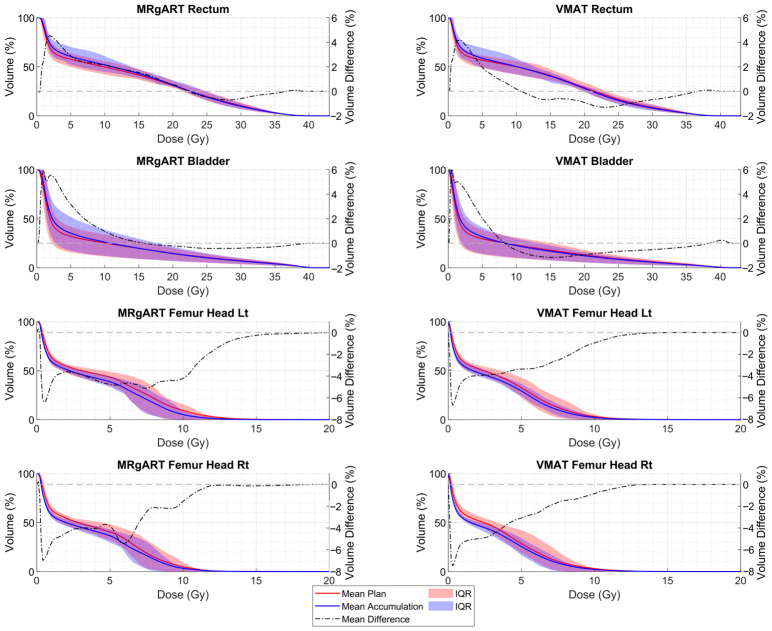
Statistical DVH curves, showing the mean plan and accumulation and their IQR for the OAR structures in the MRgART and VMAT workflows. The mean difference between the accumulated and planned doses is plotted against the right y-axis.

The MRgART dose accumulations had statistically higher prostate V_40 Gy_ and lower PTV D_0.035 mL_ than their planned doses. The VMAT dose accumulations had statistically lower PTV V_36.25 Gy_ than their planned doses. All other DVH comparisons were statistically insignificant with *p*-values > 0.0125. Table 5 shows the results of the Wilcoxon Signed Rank tests comparing the MRgART planned dose to its accumulated dose and the VMAT planned dose to its accumulated dose as well as the median DVH values across the 20 simulated treatments. Statistical significance is indicated by bold *p*-values. Figure 7 shows the planned and accumulated statistical DVH curves for the PTV and CTV in the MRgART and non-adaptive VMAT dose simulations. The statistical DVH curves are the mean and interquartile range (IQR) volumes from each dose bin. Figure 7 depicts increased target coverage and lower target hotspots for the MRgART accumulations compared to their planned doses, while the VMAT accumulations show decreased target coverage and increased target hotspots. Figure 8 shows the planned and accumulated statistical DVH curves for the OARs in both dose simulations. In terms of both MRgART and VMAT, Figure 8 shows that the low doses in the accumulations cover a larger volume of the bladder and rectum than planned, but the volumes receiving higher doses are lower than planned. For both Figure 7 and Figure 8, the mean difference between the accumulated and planned doses for each modality and structure is plotted against the right y-axis to highlight any differences.

## 4. Discussion

We evaluated the interfractional changes in volume and shape for the prostate, bladder, and rectum over the course of five fractions and how the accumulated dose differs for simulated MRgART and non-adaptive VMAT prostate SBRT treatments. The prostate tends to swell over an SBRT treatment [36,37]. This study observed a small increase in the prostate’s mean volume over fractionation, reaching a maximum volume on fraction two. An initial drop in the mean prostate volume across patients was observed in fraction one, which may be explained by some patients in this study receiving androgen deprivation therapy. There were large variations in prostate volume between patients, as the standard deviation ranged from 8.05% in fraction one to 14.3% in fraction four. The variation in prostate volume over the course of fractionation resulted in two outliers, both of which showed reduced target dose coverage in the non-adaptive VMAT plans. In one of these outlier cases, the CTV V_40 Gy_ fell out of tolerance, from 99.8% coverage in the planned dose to 88.8% coverage in the accumulation, and in the other outlier case, the CTV V_40 Gy_ dropped from 96.0% coverage in the treatment plan to 90.6% coverage in the dose accumulation.

There were no outliers in the prostate mean DICE scores, and in all three cases where the CTV DVH goal was out of tolerance, the prostate had a mean DICE score of >0.9. The case where the prostate had the lowest mean DICE score achieved primary goals for both the CTV and PTV DVH criteria. This indicates that changes in prostate volume may have a larger effect on the accumulated dose’s target coverage than the prostate shape. However, DICE measures the whole structure’s overlap and may not capture small variations. Since the planned dose drops off rapidly in the posterior direction to spare the rectum and avoid side effects such as bleeding, small anatomical shifts in this region may result in large changes in dose to the CTV and/or rectum. The instances where the bladder and rectum had outliers in DICE scores did not result in either OAR changing DVH goal categories from primary to secondary or secondary to out of tolerance. However, it is important to note that even in cases not flagged as volume or DICE score outliers, daily variations in the target or OARs’ volume or shape still occur. Such variations may cause differences between the delivered and planned doses that day and therefore affect the total dose accumulation over all fractions [37].

The dosimetric results of the simulated fractional and accumulated doses are an indicator of the clinical relevance the organ volume and shape variations have on the dose delivered over the entire course of treatment. All 100 MRgART fractional doses achieved clinical tolerance, which corresponded to all 20 MRgART dose accumulations achieving clinical tolerance. There was generally good agreement in scorecard criteria between the MRgART planned and accumulated doses, as there was only one case where an MRgART dose accumulation shifted from achieving primary goals in the reference plan to secondary goals in the accumulation. Statistical testing showed that the accumulated MRgART doses had similar OAR doses along with higher CTV coverage and lower PTV hotspots than the planned doses.

In non-adaptive VMAT, 29 of the 100 fractional doses had at least one DVH criterion outside of clinical tolerance. These variations did not always wash out over the five-fraction treatment course, as there were four cases, three in the CTV V_40 Gy_ and one in the bladder V_18.1 Gy_, where a dose criterion was out of tolerance in the accumulated dose. The non-adaptive VMAT doses did not consistently produce the same scorecard results as their planned doses, as in 13 of the 20 non-adaptive VMAT dose accumulations, a dose criterion went from achieving the primary goals in the planned dose to either achieving secondary goals or falling out of tolerance in the dose accumulation. Statistical testing showed that the accumulated VMAT doses produced lower PTV coverage than their planned doses.

The non-adaptive VMAT workflow produces higher-quality initial plans compared to the step-and-shoot MRgART workflow, with increased OAR sparing. There was no statistical difference in the dose to the PTV or CTV in the planned doses; however, the MRgART accumulations produced higher coverage in the PTV and CTV along with lower PTV hotspots. The OAR dose changed similarly between the two modalities from their planned to accumulated doses. However, since the VMAT doses produced higher-quality initial plans in terms of OAR sparing, the VMAT dose accumulations maintained greater OAR sparing compared to the MRgART accumulations. This may be due to the step-and-shoot nature of the current MRgART workflow, where the beam must shoot through portions of the OARs to reach the target. It should be noted that 19 of the 20 MRgART dose accumulations achieved the same original scorecard goals as their corresponding reference dose, meaning that the clinical goals achieved in the approved initial plan were still consistently being met. The non-adaptive VMAT workflow was able to produce better OAR sparing according to statistical testing, but there were outlier cases where the OAR doses dropped from achieving primary to secondary goals or completely fell outside of clinical tolerance. The adaptive strategy within the MRgART workflow can prevent these outlier cases form occurring.

Because the MRgART workflow allows for plan optimization each day, beam shapes can change daily with the shape of the target. This provides a dosimetric advantage, where scorecard results remain consistent with the reference dose and reduce the chance of the scorecard goals drifting out of tolerance. These dosimetric deviations were observed in the non-adaptive VMAT workflow for four plans due to the lack of plan adaptation. This study agrees with the current state of the literature, in that plan adaptations have a favorable impact on the estimated delivered dose. Xin et al. performed dose accumulations to compare MRgART to IGRT for rectal plans and found that the IGRT workflow may result in inadequate target coverage and increased bladder dose [33]. Dunlop et al. compared MRgART fractional doses with non-adaptive VMAT fractional doses for the prostate and demonstrated increased target coverage, using MRgART [20]. These studies, combined with our results, demonstrate that the MRgART workflow produces accumulated doses that more closely resemble the initially planned dose and may provide more consistent target coverage for whole prostate SBRT compared to a non-adaptive VMAT workflow, with a median accumulated CTV_V40 Gy_ difference of 2.48%. This study further adds to the existing literature by incorporating an estimated dose accumulation over the full SBRT treatment course, demonstrating that observed dose discrepancies in a non-adaptive workflow do not always wash out over multiple fractions.

We acknowledge several limitations of this work. This study provides an estimate of the total dose accumulated over a five-fraction SBRT, and only the interfractional anatomical motion is accounted for. The anatomy was assumed to be static over the course of treatment delivery, and the intrafractional motion was ignored. The intrafractional motion may impact MRgART more than the non-adaptive VMAT workflow due to longer treatment times; however, the use of intrafraction cine MRI and tumor tracking can mitigate this issue. Future work could use the live tracking of the tumor to get a simulated dose accumulation closer to the actual dose delivered. When registering the non-adaptive VMAT beams to each daily image, only 3 DOF shifts were performed. Clinics practicing non-adaptive prostate SBRT may have access to a 6 DOF couch, allowing for rotational couch corrections, which may allow for improved alignment. This limitation is minor because, in our clinical practice, immobilization devices are used to ensure reproducible patient setup, and if rotations are observed, the patient is realigned manually. The dose accumulations were performed using DIR to transfer the doses to a single fraction, so deviations in the doses may be partially due to the inaccuracies of the DIR. Care was taken to limit the dosimetric deviations caused by the DIR, but uncertainties inherent to DIR algorithms still exist [38]. The results of this study may be influenced by the optimization parameters set in the TPS, particularly for the daily plans in the MRgART workflow, where the optimization iterations were minimized to reflect the time constraints found in the online workflow. The optimization parameters used in this study are standard parameters being used at Mary Bird Perkins Cancer Center. Only whole prostate SBRT cases were considered in this study. Since MRgART requires more time and resources than traditional treatment methods, future work should compare dose accumulations in MRgART with other modalities, such as conventional fractionation schemes across other treatment sites, to identify which sites may benefit the most from plan adaptation. The use of pseudo-CT datasets for dose calculation may introduce additional uncertainty into the dose calculations. However, this is a minor limitation because any uncertainty would apply to both the adaptive and conventional dose accumulations. Additionally, the pseudo-CT datasets were generated using a commercially available software package with FDA 510(k) clearance that has been commissioned for clinical treatment planning at our institution. Finally, the lack of measured dosimetric data as well as clinical outcome data is another limitation of this study. Such methods and results are outside the scope of this work.

## 5. Conclusions

We successfully evaluated how the interfractional motion of the internal anatomy affects the total simulated dose over a five-fraction, whole prostate SBRT treatment regime and compared how these effects differ in MRgART ATS and non-adaptive VMAT workflows. There was reduced target coverage between the planned and accumulated doses for the non-adaptive VMAT plan simulations, and four of the 20 VMAT dose accumulations fell outside of clinical tolerance. All MRgART dose accumulations remained within clinical tolerance for all DVH goals. Although the non-adaptive VMAT workflow can generate high-quality plans and achieve favorable dose simulations, the MRgART ATS workflow’s ability to adapt the plan to the daily anatomy ensures dosimetric consistency in the simulated dose. This work provides clinicians with a stronger estimate of the total dose received by the patient for both workflows and demonstrates the dosimetric advantages that occur in the MRgART.

## Figures and Tables

**Table 1:** Primary and secondary goals for all simulated SBRT plans.

Structure	Constraint	Primary Goal	Secondary Goal
PTV	V_36.35 Gy_	≥98%	≥95%
PTV	D_0.035 mL_	≤43.5Gy	≤44Gy
CTV	V_40 Gy_	≥95%	≥90%
Rectum	D_0.5 mL_	≤38Gy	≤41Gy
Rectum	V_36 Gy_	≤1mL	≤3mL
Rectum	V_32.6 Gy_	≤10%	≤15%
Rectum	V_29 Gy_	≤20%	≤25%
Rectum	V_18.1 Gy_	≤50%	≤55%
Bladder	V_37 Gy_	≤10mL	≤20mL
Bladder	V_18.1 Gy_	≤40%	≤45%
Femur Head Lt	V_14.5 Gy_	≤5%	≤10%
Femur Head Lt	V_D1 mL_	≤19.9Gy	≤22Gy
Femur Head Rt	V_14.5 Gy_	≤5%	≤10%
Femur Head Rt	V_D1 mL_	≤19.9Gy	≤22Gy

**Table 2:** Mean and standard deviation DICE scores and MDA for DIR QA.

Contour	Mean DICE	σ DICE	Mean MDA	σ MDA
Bladder	0.966	0.021	0.687	0.558
Femur Head Lt	0.974	0.008	0.367	0.127
Femur Head Rt	0.974	0.007	0.351	0.114
PTV	0.949	0.017	0.606	0.233
Prostate	0.940	0.020	0.564	0.214
Rectum	0.900	0.036	0.968	0.462

**Table 3:** Mean and standard deviation dose differences in the transferred dose for DIR QA. PTV D_0.03 mL_ was normalized to 40 Gy to get the % difference.

Structure	Constraint	Mean Difference MRgART	σ MRgART	Mean Difference VMAT	σ VMAT
PTV	V_36.25 Gy_	0.17%	0.27%	0.38%	0.67%
PTV	D_0.03 mL_	0.10%	0.07%	0.09%	0.07%
Rectum	V_36 Gy_	0.04%	0.29%	0.06%	0.32%

**Table 4:** Results of Wilcoxon Signed Rank tests comparing DVH criteria of MRgART planned doses to VMAT planned doses and MRgART dose accumulations to VMAT dose accumulations. Bold *p*-values indicate statistical significance < 0.0125, with Bonferroni correction.

Structure	Constraint	Median MRgART Plan	Median VMAT Plan	*p*-Value	Median MRgART Accumulation	Median VMAT Accumulation	*p*-Value
PTV	V_36.35 Gy_	100.00	99.98	0.0167	100.00	99.31	**0.0003**
PTV	D_0.035 mL_	41.82	41.81	0.3241	41.43	41.87	**0.0001**
CTV	V_40 Gy_	98.16	98.13	0.5628	98.94	96.46	**0.0048**
Rectum	D_0.5 mL_	37.52	37.28	0.0145	37.60	37.21	0.4008
Rectum	V_36 Gy_	1.33	1.15	**0.0006**	1.42	1.18	0.1305
Rectum	V_32.6 Gy_	7.38	5.05	**0.0014**	6.25	4.42	0.0136
Rectum	V_29 Gy_	14.72	9.52	**0.0002**	11.82	8.48	**0.0010**
Rectum	V_18.1 Gy_	36.87	32.91	0.0894	36.88	33.81	**0.0004**
Bladder	V_37 Gy_	4.82	3.85	**0.0002**	4.96	3.30	0.0290
Bladder	V_18.1 Gy_	17.41	14.68	**<0.0001**	14.31	12.75	**0.0020**
Femur Head Lt	V_14.5 Gy_	0.00	0.00	0.1056	0.00	0.00	0.5896
Femur Head Lt	V_D1 mL_	11.46	9.96	**0.0000**	11.28	9.93	**0.0012**
Femur Head Rt	V_14.5 Gy_	0.00	0.00	1.0000	0.00	0.00	1.0000
Femur Head Rt	V_D1 mL_	10.23	8.88	**0.0021**	10.4	9.00	0.0328

**Table 5:** Results of Wilcoxon Signed Rank tests comparing DVH criteria of MRgART planned doses to MRgART dose accumulations and VMAT planned doses to VMAT dose accumulations. Bold *p*-values indicate statistical significance < 0.0125, with Bonferroni correction.

Structure	Constraint	Median MRgART Plan	Median MRgART Accumulation	*p*-Value	Median VMAT Plan	Median VMAT Accumulation	*p*-Value
PTV	V_36.35 Gy_	100.00	100.00	0.7550	99.98	99.31	**0.0011**
PTV	D_0.035 mL_	41.82	41.43	**0.0001**	41.81	41.87	0.8082
CTV	V_40 Gy_	98.16	98.94	**0.0012**	98.13	96.46	0.0192
Rectum	D_0.5 mL_	37.52	37.60	0.8695	37.28	37.21	0.6813
Rectum	V_36 Gy_	1.33	1.42	0.2512	1.15	1.18	0.6742
Rectum	V_32.6 Gy_	7.38	6.25	0.0583	5.05	4.42	0.3118
Rectum	V_29 Gy_	14.72	11.82	0.1650	9.52	8.48	0.2611
Rectum	V_18.1 Gy_	36.87	36.88	0.4524	32.91	33.81	0.8695
Bladder	V_37 Gy_	4.82	4.96	0.3118	3.85	3.30	0.1893
Bladder	V_18.1 Gy_	17.41	14.31	0.7841	14.68	12.75	0.7841
Femur Head Lt	V_14.5 Gy_	0.00	0.00	0.3700	0.00	0.00	0.3711
Femur Head Lt	V_D1 mL_	11.46	11.28	0.5706	9.96	9.93	0.0594
Femur Head Rt	V_14.5 Gy_	0.00	0.00	1.0000	0.00	0.00	1.0000
Femur Head Rt	V_D1 mL_	10.23	10.40	0.4749	8.88	9.00	0.3300

## Data Availability

The original contributions presented in this study are included in the article/Supplementary Material. Further inquiries can be directed to the corresponding authors.

## Supplementary Materials

The following supporting information can be downloaded at https://www.mdpi.com/article/10.3390/cancers18091417/s1, Table S1: Per patient per contour DIR QA data, containing DICE scores, MDA, and their corresponding σ. Table S2: Dosimetric difference data from DIR for all scorecard DVH points.

## Abbreviations

The following abbreviations are used in this manuscript:

ATS	Adapt To Shape
CBCT	Cone Beam Computed Tomography
CT	Computed Tomography
DIR	Deformable Image Registration
DOF	Degree of Freedom
DVH	Dose Volume Histogram
FFF	Flattening Filter-Free
IGRT	Image-Guided Radiation Therapy
IMRT	Intensity-Modulated Radiation Therapy
IQR	Interquartile Range
LINAC	Linear Accelerator
MLC	Multileaf Collimator
MRI	Magnetic Resonance Imaging
MRgART	Magnetic Resonance-Guided Adaptive Radiation Therapy
OAR	Organ At Risk
PTV	Planning Target Volume
RT	Radiation Therapy
SBRT	Stereotactic Body Radiation Therapy
TPS	Treatment Planning System
VMAT	Volumetric Modulated Arc Therapy
